# A72 THE ENDOSCOPIC YIELD OF PRIORITIZED INDICATIONS USING A PROVINCIAL-WIDE COLONOSCOPY REFERRAL FORM

**DOI:** 10.1093/jcag/gwad061.072

**Published:** 2024-02-14

**Authors:** A Barkun, K Ravanbakhsh, D Kim, G Milky, P Stanowski, O Geraci, M Martel, C Menard, D von Renteln

**Affiliations:** McGill University Health Centre, Montreal, QC, Canada; Research Institute of the McGill University Health Centre, Montreal, QC, Canada; McGill University Health Centre, Montreal, QC, Canada; Research Institute of the McGill University Health Centre, Montreal, QC, Canada; Research Institute of the McGill University Health Centre, Montreal, QC, Canada; Research Institute of the McGill University Health Centre, Montreal, QC, Canada; Research Institute of the McGill University Health Centre, Montreal, QC, Canada; Universite de Sherbrooke, Sherbrooke, QC, Canada; Centre Hospitalier de l'Universite de Montreal, Montreal, QC, Canada

## Abstract

**Background:**

The widespread use of a standardized and validated province-wide colonoscopy referral form (PCRF), regrouping mutually exclusive indications into suggested priority wait time categories, has allowed for a more comprehensive assessment of routine colonoscopy practice

**Aims:**

To validate the Quebec PCRF by better characterizing endoscopic finding yields according to specific clinical indications.

**Methods:**

This retrospective cohort study includes consecutive adult patients with available data from the PCRF from two tertiary hospitals. The primary outcome was the diagnostic rates of colorectal cancer (CRC). Secondary outcomes assessed incidences of clinically significant lesions (CSL) on endoscopy and confirmed at pathology. These are CRC, advanced adenomas, sessile serrated polyps, polyps ≥ 5mm, colitis, colonic strictures, and miscellaneous other findings excluding hemorrhoids and diverticulosis. Descriptive and inferential statistics, and multivariable regression predictive modeling are reported.

**Results:**

Overall, 14,657 patients (mean age 59.2 ± 14.0 years, 50.9% female) were included from September 2018 to August 2022. The most frequent indications for colonoscopy were polyp surveillance (**IN13,** 20.8%), recent change in bowel habits (**IN7,** 11.9%), a positive fecal immunochemical test (**IN5,** 8.3%), a family history of CRC or polyps (first-degree relative) (**IN8**, 6.4%), and suspected active inflammatory bowel disease (IBD) (**IN3**, 5.8%). Overall, 40.6% had CSL, including CRCs in 0.8%. CRCs were found more frequently for **IN2** (19.7% vs 2.0%, pampersand:003C0.01), **IN5** (29.9% vs 8.2%, pampersand:003C0.01), and an unexplained documented iron deficiency anemia (**IN6**) (11.8% vs 5.2%, pampersand:003C0.01). CSLs were more frequent for **IN3** (1.6% vs 5.8%, p=0.04), **IN7** (4.7% vs 12.0%, p=0.01), IN8 (0.8% vs 6.5%, p=0.01), **IN13** (7.1% vs 20.9%, pampersand:003C0.01), IBD surveillance **(IN15,** 0.0% vs 4.0%, p=0.02), and surveillance for a significant family history (**IN21**, 0.0% vs 3.7%, p=0.02).

On multivariable analysis, CRC was significantly associated with IN2 OR=31.8 (4.86; 208.7), IN5 OR=19.2 (3.86; 95.7), and IN6 OR=15.7 (2.37; 78.0) - (all are high priority PCRF referrals with a suggested shorter wait time than for most other indications). CSLs were significantly associated with age (OR=1.03 (1.02; 1.03)), **IN2** (OR=2.32 (1.35; 3.99)), **IN5** (OR=2.37 (1.74; 3.22)), and **IN13** (OR= 1.66 (1.36; 2.02)).

**Conclusions:**

This large cohort study confirms the validity of indications and corresponding prioritization of wait times of a PCRF.

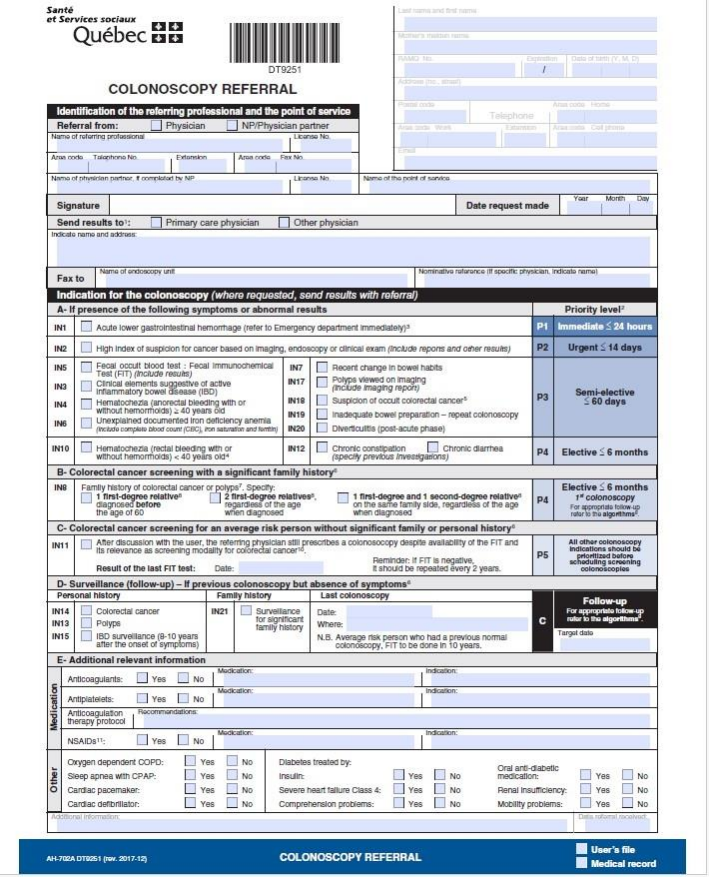

**Funding Agencies:**

CPAC and MSSS

